# Alterations of optic tract and retinal structure in patients after thalamic stroke

**DOI:** 10.3389/fnagi.2022.942438

**Published:** 2022-07-28

**Authors:** Chen Ye, William Robert Kwapong, Wendan Tao, Kun Lu, Ruosu Pan, Anmo Wang, Junfeng Liu, Ming Liu, Bo Wu

**Affiliations:** Department of Neurology, West China Hospital, Sichuan University, Chengdu, China

**Keywords:** thalamic stroke, retinal structure, optic tract, retrograde degeneration, MRI, OCT

## Abstract

**Objectives:**

To investigate the association between degeneration of retinal structure and shrinkage of the optic tract in patients after thalamic stroke.

**Materials and methods:**

Patients with unilateral thalamic stroke were included. Structural magnetic resonance imaging (MRI) and optical coherence tomography (OCT) were performed to obtain parameters of optic tract shrinkage (lateral index) and retina structural thickness (retinal nerve fiber layer, RNFL; peripapillary retinal nerve fiber layer, pRNFL; ganglion cell-inner plexiform layer, GCIP), respectively. Visual acuity (VA) examination under illumination was conducted using Snellen charts and then converted to the logarithm of the minimum angle of resolution (LogMAR). We investigated the association between LI and OCT parameters and their relationships with VA.

**Results:**

A total of 33 patients and 23 age-sex matched stroke-free healthy controls were enrolled. Patients with thalamic stroke showed altered LI compared with control participants (*P* = 0.011) and a significantly increased value of LI in the subgroup of disease duration more than 6 months (*P* = 0.004). In these patients, LI were significantly associated with pRNFL thickness (β = 0.349, 95% confidence interval [CI]: 0.134–0.564, *P* = 0.002) after adjusting for confounders (age, sex, hypertension, diabetes, dyslipidemia, and lesion volume). LI and pRNFL were both significantly associated with VA in all patients (LI: β = −0.275, 95% CI: −0.539 to −0.011, *P* = 0.041; pRNFL: β = −0.023, 95% CI: −0.046 to −0.001, *P* = 0.040) and in subgroup of disease duration more than 6 months (LI: β = −0.290, 95% CI: −0.469 to −0.111, *P* = 0.002; pRNFL: β = −0.041, 95% CI: −0.065 to −0.017, *P* = 0.003).

**Conclusion:**

Shrinkage of the optic tract can be detected in patients with thalamic stroke, especially after 6 months of stroke onset. In these patients, the extent of optic tract atrophy is associated with pRNFL thickness, and they are both related to visual acuity changes.

## Introduction

The last few decades have seen a substantial increase in research identifying alterations in retinal structure and microvasculature after stroke (Park et al., 2013; Wang et al., 2014; Zhang et al., 2020; Kwapong et al., 2021). It is suggested that the retina can act as a novel and non-invasive imaging marker with potential diagnostic and clinical significance in neurological diseases (Cabrera DeBuc et al., 2017; Mutlu et al., 2017; Kwapong et al., 2018). Thinning of the retinal nerve fiber layer (RNFL) and ganglion cell layer (GCL)-inner plexiform layer (GCIPL) has been consistently documented in previous reports (Park et al., 2013; Zhang et al., 2020; Kashani et al., 2021; Snyder et al., 2021; Ye et al., 2022) in most cerebrovascular disorders. These reports suggest retinal neurodegeneration reflects cerebral neurodegeneration, and this mechanism may occur simultaneously. Importantly, prior reports (Ong et al., 2015; Mutlu et al., 2017; Chua et al., 2021) suggested that the retinal sublayer thicknesses reflect the cerebral microstructure (white matter and gray matter microstructure). The brain and retina share many characteristics, including similar microvasculature, embryology, and precise neuronal cell layers; axons from the optic nerve form a direct link, through the optic head, between the retina and the brain, thus, it is suggested that damage in the brain is associated with retinal thinning (especially for peripapillary RNFL and macular GCL) (Cabrera DeBuc et al., 2017; Kwapong et al., 2018; Mutlu et al., 2018).

As the central hub of the brain, the thalamus plays an important role in relaying sensory information to and from the cerebral cortex (Bogousslavsky et al., 1988). Notably, projections from the visual cortex are *via* the thalamus, emphasizing its importance in vision. Thalamic stroke accounts for 3–4% of ischemic stroke and is associated with neuro-ophthalmic deficits (Bogousslavsky et al., 1988; Adams<suffix>Jr.</suffix>, Bendixen et al., 1993; Schmahmann, 2003). Impaired visual acuity and oculomotor deficits have been reported in a series of studies (Adams<suffix>Jr.</suffix>, Bendixen et al., 1993; Rorden and Brett, 2000; Weidauer et al., 2004; Moon et al., 2021). Notably, neuro-ophthalmic deficits after a thalamic injury can also lead to a great disease burden and serious disability affecting everyday activities (Moon et al., 2021). Previous reports focused on cerebral changes associated with thalamic stroke (Chen et al., 2016; Conrad et al., 2022), while very less is known about the retinal changes and their association with the cerebral changes.

Magnetic resonance imaging (MRI) studies conducted on visual system-damaged subjects have revealed objective signs of shrinkage of optic tract (OT) volume and denoted it as the lateral index (LI) (Bridge et al., 2011; Cowey et al., 2011; Millington et al., 2014). The degeneration of OT, known as transsynaptic retrograde degeneration (TRD), may have an impact on the recovery effect from visual restoration training in cortical blindness subjects and indicates interindividual variability of relatable therapy (Fahrenthold et al., 2021). As the thalamus is the relay center and is involved in visual processing, we hypothesize that damage to the thalamus may result in the disruption of connections in the visual tract, especially the optic tract (OT), which may cause retrograde degeneration of the optic nerve, resulting in retinal changes. Exploring the correlations between retinal thicknesses and brain structural changes in thalamic stroke may give insights into the mechanism underlying visual disturbances and neurodegeneration during the disease cascade and ultimately benefit treatments for these patients.

Therefore, this study aimed to investigate the association between retinal thickness and structural OT indicators in thalamic stroke, and we further explored their relationships with clinical visual features in these patients.

## Materials and methods

### Study population

Patients with first-ever unilateral thalamic stroke who visited the Department of Neurology, West China Hospital, Sichuan University, were consecutively enrolled from 2020 to 2022. During the same study period, age-sex matched healthy control participants who had no history of neurological diseases and vascular risks were recruited from voluntary persons dwelling in native communities. Patients were included in the study if they (1) had a clinical diagnosis of first-ever unilateral thalamic stroke confirmed by experienced neurologists and MRI examinations; (2) completed an OCT retinal imaging scan and a structural brain MRI examination; and (3) provided written informed consent. The exclusion criteria of our patients were as follows: (1) diagnosed with diabetic retinopathy or other retinal diseases; (2) glaucoma; (3) pacemaker or other contraindications for MRI examinations; (4) a history of stroke or any other pathological conditions of neurological diseases; and (5) poor MRI or OCT imaging qualities. Control participants were included if they met the following criteria: (1) aged 18 years or older; (2) could undergo and cooperate with retinal and MR imaging; (3) had no history of cerebrovascular diseases, neurodegenerative diseases, or any other kind of central nervous system illness; and (4) no history of retinal diseases or ophthalmic abnormalities that could affect the retinal structure/microvasculature.

Demographic and clinical information were collected in a standardized format, including sex, age, and risk factors for cerebrovascular disease (history of hypertension, diabetes, and dyslipidemia). Time since stroke onset (months) and National Institute of Health Stroke Scale (NIHSS) scores were also documented. Since it has been well recognized that the process of TRD is time-dependent (usually occurring significantly from 3 to 7 months to the first few years maximally) (Jindahra et al., 2012), along with the expansion course of poststroke visual deficit, which becomes steady after 6 months (Cavanaugh and Huxlin, 2017; Wang et al., 2017; Saionz et al., 2020), in the following analysis we divided our patients into two subgroups: Group 1 (≤ 6 months) and Group 2 (>6 months). Visual acuity (VA) under illumination was completed for each eye using Snellen charts by an experienced neuro-ophthalmologist (WK) and later converted to the logarithm of the minimum angle of resolution (LogMAR). Standardized neurological examinations, including eye movement and visual field, were conducted by senior neurology residents (CY and KL) under the guidance of an experienced neurologist (BW). Written informed consent was obtained from each participant or their legal guardians, and approval of our project was obtained from the Ethics Committee of West China Hospital of Sichuan University [No. 2020 (922)].

### Magnetic resonance imaging acquisition and obtaining of optic tract volume and lateral index within each subject

All participants enrolled in this study underwent structural MRI examination (3D-T1 weighted image, brain volume sequence, and BRAVO) using a 3.0 T MR scanner (SIGNA™ Premier, GE Medical Systems) with the 48-channel head coil, with parameters as follows: repetition time (TR)/echo time (TE) = 7.2/3.0 ms; field of view = 256 × 256 mm; matrix = 256 × 256; slice thickness = 1.0 mm, a total of 152 slices with no gap; flip angle = 12°. Routine clinical sequences including diffusion-weighted images (DWI), T2-weighted images, and fluid-attenuated inversion recovery (FLAIR) images were also acquired. Head motion and scanner noise was reduced using comfortable foam padding and earplugs.

**FIGURE 1:**
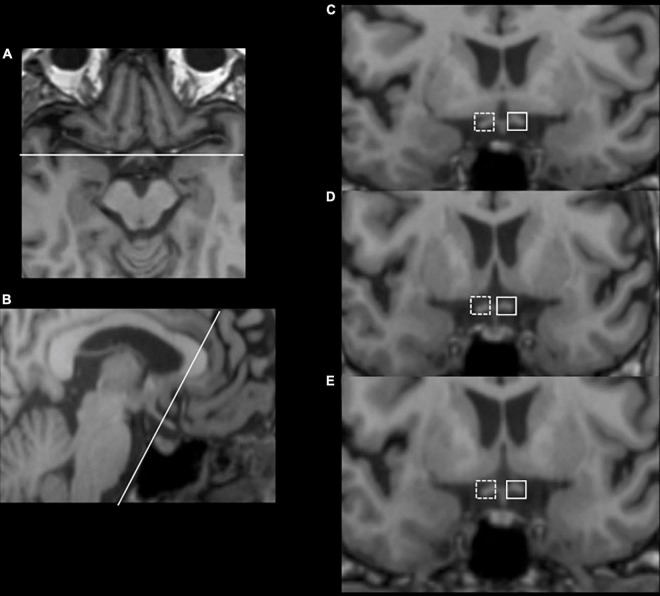
Illustration of optic tract measurements with an example from a 56-year-old female thalamus stroke patient. Consecutive **(C–E)** coronal slices, which have been resliced and reoriented to be perpendicular to the OT **(B)**, were obtained starting with three slices posterior to the beginning of the optic chiasm **(A)**. Equal-sized masks were hand-drawn on the two sides of optic tracts in each slice, with the solid border as the contralateral side and the dashed border as the ipsilateral side.

Optic tract volume (OT) measurements and lateral index (LI) calculating methods were adapted from previously published reports (Bridge et al., 2011; Millington et al., 2014; Fahrenthold et al., 2021) based on the intensity values of the T1-weighted images. First, structural MRI images were reoriented and resampled in standard space (1 mm) using FMRIB software library (FSL) image analysis software^1^ to make the optic tract parallel to the anterior-posterior axis so that the effects of individual head orientations in the scanner were minimized. Second, equal-sized masks were hand-drawn on the two sides of optic tracts in each slice of processed MRI images (planes parallel and perpendicular to the OT), starting from the third slice posterior to the origin of the optic chiasm and continuing until the OT was unable to be discerned from surrounding tissues, as shown in Figure 1. The ipsilateral OT (iOT) and contralateral OT (cOT) were stratified according to the location of the stroke lesion in the patient group, while it was described as right or left OT (rOT and lOT) in the control group. Third, under Fahrenthold et al.’s study (Fahrenthold et al., 2021), we built an intensity threshold filter, ranging from 5 to 95% of the maximal brightness values of T1-weighted signals, to obtain the number of voxels in the masks as the OT volume. Then we performed the calculation of each subject’s lateral index (LI) using the following formulas: LI = (iOT–cOT)/(iOT + cOT) in patients and LI = (rOT–lOT)/(rOT + lOT) in controls. By doing so, only voxels containing most of the white matter were included in the analysis.

We also measured the stroke lesion volume by drawing lesion masks manually on the structural MR images with the combination of DWI and FLAIR images using MRIcron (Rorden and Brett, 2000). Then lesion volumes were obtained by the volume of interest (VOI, cm^3^). Two trained neurologists (C.Y. and W.D.T.) were engaged in the MRI processing and measurements blinded to clinical information, and an experienced neurologist (B.W.) was consulted when disagreement occurred. The inter-rater agreement was good with an intraclass correlation coefficient (ICC) of 0.80 for LI and 0.83 for lesion volume.

### Optical coherence tomography examination

**FIGURE 2:**
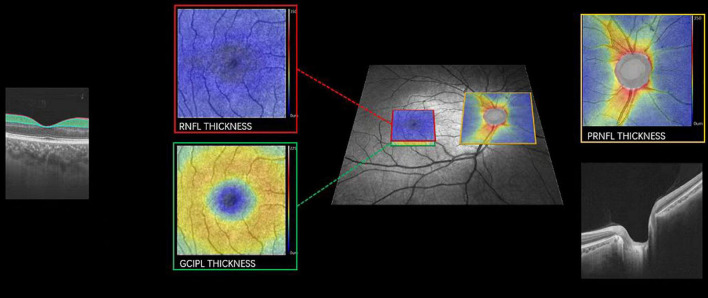
Imaging of pRNFL, RNFL, and GCIPL thickness. Representative image focusing on the macula (red and green box) and optic nerve (yellow box) with the corresponding cross-sectional view of the retina. pRNFL, peripapillary retinal nerve fiber layer; RNFL, retinal nerve fiber layer; GCIPL, the ganglion cell and inner plexiform layer. A color map of the OCT structural thickness was shown to indicate the thickness. Warm colors indicate a thick structure, while cold colors indicate a thin structure.

Swept-source optical coherence tomography (SS-OCT, VG 200, SVision Imaging Limited, Luoyang, China) was used to image the structure of the retina by an experienced neuro-ophthalmologist (WK). The specifications of the OCT tool have been well described in our previous report (Ye et al., 2022). As shown in Figure 2, the peripapillary retinal nerve fiber layer (pRNFL) was done using the optic nerve head protocol with a scanning range covering a circle with a diameter of 3.45 mm focused on the optic disc. Structural OCT imaging of the macula was done with 18 radial scan lines focused on the fovea. Automatic segmentation of the retinal nerve fiber layer (RNFL) and ganglion cell-inner plexiform layer (GCIPL) was done with a built-in algorithm in the OCT tool. Average thicknesses of the RNFL (measured in μm) and GCIPL (measured in μm) in a 3 × 3 mm area around the fovea were used in this study.

### Statistical analysis

Continuous variables with a normal distribution were expressed as mean ± standard deviation (SD), while those with skewed distribution were expressed as medians and interquartile ranges (IQR). Categorical variables are presented as frequencies and percentages. Participants’ demographic and clinical variables were assessed using a chi-square test for categorical variables and an independent sample *t*-test or non-parametric test for continuous variables. Multilinear regression was performed to assess the associations among OT parameters and retina structural parameters, and VA, LogMAR while adjusting for confounding factors (age, sex, hypertension, diabetes, dyslipidemia, and lesion volume) with β coefficient and 95% confidence interval (95% CI). All data were analyzed using SPSS (version 23; SPSS, Chicago, United States) and GraphPad Prism (version 9.3.0; GraphPad Software, San Diego, United States), and a two-sided *P* < 0.05 was considered statistically significant.

## Results

### Baseline demographic and clinical characteristics

A total of 33 patients with first-ever thalamic stroke (27 men, mean age = 59.8 ± 10.7 years) and 23 age-sex matched healthy control participants (17 men, mean age = 57.9 ± 7.5 years) were enrolled in this study. In the thalamic stroke group, 19 patients had a history of hypertension, 10 had diabetes, and 5 had dyslipidemia. The baseline demographic and clinical characteristics are shown in Table 1. None of the control participants had a history of hypertension, diabetes, and dyslipidemia. For stroke type, 32 had an ischemic stroke while 3 had a hemorrhagic stroke; 18 patients had right-sided stroke lesions and 17 were left-sided. The median NIHSS score of patients with thalamic stroke was 1 (IQR, 1–2), and the median disease duration since stroke onset was 7.0 (IQR, 0.2–44) months, of which 13 (37.1%) were less than or equal to 6 months and 22 (62.9%) were greater than 6 months. No significant visual fields and oculomotor deficits were observed in this study, but the visual acuity (VA, LogMAR) of the patients’ group was worse than that of control participants (0.24 ± 0.21 vs. 0.01 ± 0.08, *P* < 0.001). The stroke lesion volume was 0.14 (IQR, 0.06–0.54) cm^3^. There was no significant difference (P = 0.052) in the visual acuity between patients with left thalamic stroke (0.26 ± 0.21) and patients with right thalamic stroke (0.17 ± 0.14).

**TABLE 1:** Baseline demographic and clinical characteristics of all included subjects.

Characteristics	Thalamic stroke (*n* = 35)	Controls (*n* = 23)	*P*
Age, years	59.8 ± 10.7	57.9 ± 7.5	0.449
Gender (males), n (%)	27 (77.1)	17 (73.9)	0.779
Hypertension, n (%)	19 (54.2)	–	
Diabetes, n (%)	10 (28.6)	–	
Dyslipidemia, n (%)	5 (14.3)	–	
Stroke type		–	
Ischemic, n (%)	32 (91.43)		
Hemorrhagic, n (%)	3 (8.57)		
Lesion location		–	
Left, n (%)	17 (48.57)		
Right, n (%)	18 (51.43)		
Duration, months	7.0 (0.2 to 44)	–	
≤ 6 months, n (%)	13 (37.1)	–	
>6 months, n (%)	22 (62.9)	–	
NIHSS score	1 (1 to 2)	–	
Lesion volume, cm^3^	0.14 (0.06 to 0.54)	–	
VA, LogMAR	0.24 ± 0.21	0.01 ± 0.08	** < 0.001**

NIHSS, National Institute of Health Stroke Scale; VA: visual acuity. Values in bold indicate *P* < 0.05.

All the patients underwent SS-OCT imaging and 67 eyes (33 eyes on the ipsilateral side and 34 eyes on the contralateral side; three eyes were excluded due to poor cooperation and bad image quality) were included in this study, divided into two groups based on the disease duration: Group 1, ≤ 6 months; and Group 2, >6 months. No significant differences were found in the retinal structural parameters (pRNFL, RNFL, and GCIPL thickness) and VA between Group 1 and Group 2 (all *P*>0.05). Details are displayed in Supplementary Table 1.

### Optic tract parameters in patients with thalamic stroke and comparisons to control participants

**FIGURE 3:**
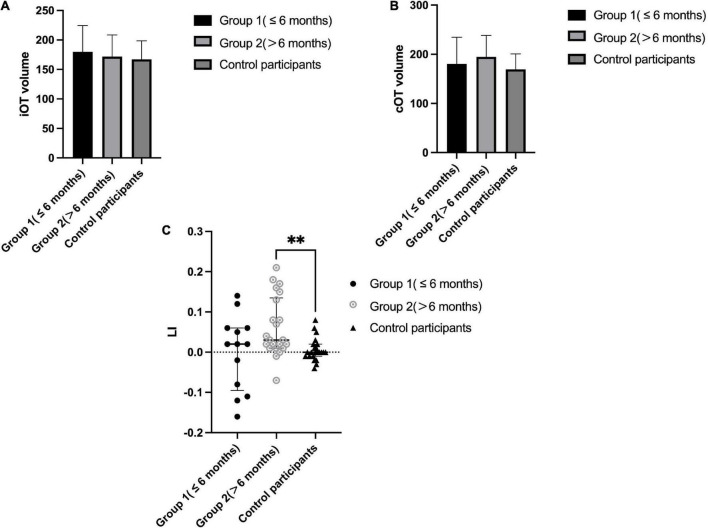
Optic tract parameters in patients with thalamic stroke and comparison to control participants. **(A)**: Ipsilateral OT volume (voxel numbers) in Group 1 (≤ 6 months) and Group 2 (>6 months) patients and control participants (right side); **(B)**: Contralateral OT volume (voxel numbers) in Group 1 (≤ 6 months) and Group 2 (>6 months) patients and control participants (left side); **(C)**: LI in Group 1 (≤ 6 months) and Group 2 (>6 months) patients and control participants; ^**^P < 0.05.

As shown in Figure 3 (details in Supplementary Table 2), ipsilateral optic tract volume (iOT) was 189.51 ± 47.51 and the contralateral side (cOT) was 174.80 ± 39.18 in patients with thalamic stroke, while right-side optic tract volume was 169.26 ± 31.49 and the left side was 167.17 ± 31.30 in control participants. No significant differences were found when comparing patients to control participants (all *P*>0.05). Optic tract lateral index (LI) differed significantly among these different groups (*P* = 0.006). When compared with control participants (0.002, IQR −0.006 to 0.015), all thalamic stroke groups showed a prominently increased LI value of 0.021 (IQR, 0.007 to 0.078) with *P* = 0.011. Notably, the LI of Group 2 (0.031, IQR 0.01–0.135) was significantly greater than that in the control group (*P* = 0.004). Although not reaching statistical significance (*P* = 0.183), Group 1 showed a decreased median LI value (0.015, IQR −0.091 to 0.61) compared with Group 2.

### Associations between LI and retina structural parameters in patients with thalamic stroke

Associations between LI and retina structural parameters (pRNFL, RNFL, and GCIPL thickness) were assessed in patients with thalamic stroke, as shown in Table 2. After adjusting for confounders (age, gender, vascular risk factors [i.e., hypertension, diabetes, and dyslipidemia], and lesion volume), LI had no significant association with pRNFL, RNFL, and GCIPL (all *P*>0.05) in the patients’ group and Group 1 (≤ 6 months). In patients with duration >6 months (Group 2), LI was significantly associated with pRNFL (β = 0.349, 95% CI 0.134–0.564, *P* = 0.002). However, no statistically significant associations were found in RNFL (*P* = 0.962) and GCIPL (*P* = 0.917) with LI in this group.

**TABLE 2:** Associations between LI and retina structural parameters in patients with thalamic stroke.

	All			Group 1 (≤ 6 months)			Group 2 (>6 months)		
	β	95% CI	*P*	β	95% CI	*P*	β	95% CI	*P*
pRNFL, μm	0.238	−0.028 to 0.505	0.078	0.028	−0.095 to 0.151	0.626	0.349	0.134 to 0.564	**0.002**
RNFL, μm	−0.009	−0.272 to 0.255	0.948	−0.077	−0.233 to 0.079	0.297	−0.005	−0.209 to 0.199	0.962
GCIPL, μm	0.038	−0.225 to 0.301	0.775	−0.065	−0.271 to 0.141	0.499	0.011	−0.201 to 0.223	0.917

Adjusted for age, gender, vascular risks (hypertension, diabetes, and dyslipidemia), and lesion volume. pRNFL, peripapillary retinal nerve fiber layer; RNFL, retinal nerve fiber layer; GCIPL, the ganglion cell and inner plexiform layer. Values in bold indicate *P* < 0.05.

### Associations of LI and retina structural parameters with visual acuity, logarithm of the minimum angle of resolution (LogMAR) in patients with thalamic stroke

We further explored the associations of LI and retinal structural parameters with visual acuity (VA, LogMAR) in patients with thalamic stroke, respectively. As shown in Table 3, LI and pRNFL thickness were both significantly correlated with VA in all patients after adjusting for confounding factors (LI: β = −0.275, 95% CI −0.539 to −0.011, *P* = 0.041; pRNFL: β = −0.023, 95% CI −0.046 to −0.001, *P* = 0.040). Particularly, in Group 2 of disease duration of more than 6 months, these adjusted associations remained statistically significant (LI: β = −0.290, 95% CI −0.469 to −0.111, *P* = 0.002; pRNFL: β = −0.041, 95% CI −0.065 to −0.017, *P* = 0.003).

**TABLE 3:** Associations between LI and retina structural parameters with VA, LogMAR in patients with thalamic stroke, respectively.

	All			Group 1 (≤ 6 months)			Group 2 (>6 months)		
	β	95% CI	*P*	β	95% CI	*P*	β	95% CI	*P*
LI	−0.275	−0.539 to −0.011	**0.041**	0.086	−0.170 to 0.342	0.470	−0.290	−0.469 to −0.111	**0.002**
pRNFL, μm	−0.023	−0.046 to −0.001	**0.040**	0.001	−0.075 to 0.077	0.968	−0.041	−0.065 to −0.017	**0.003**
RNFL, μm	−0.010	−0.217 to 0.197	0.923	−0.276	−0.868 to 0.317	0.286	0.068	−0.196 to 0.333	0.588
GCIPL, μm	−0.017	−0.053 to 0.019	0.334	−0.030	−0.151 to 0.092	0.557	−0.013	−0.058 to 0.032	0.547

Adjusted for age, gender, vascular risks (hypertension, diabetes, and dyslipidemia), and lesion volume. VA: visual acuity, LogMAR. pRNFL: peripapillary retinal nerve fiber layer; RNFL: retinal nerve fiber layer; GCIPL: the ganglion cell and inner plexiform layer. Values in bold indicate *P* < 0.05.

## Discussion

Our current report builds upon our previous report on retinal neurodegeneration following thalamic infarction (Ye et al., 2022) and the fundamental observations that such retrograde transsynaptic neurodegeneration after visual pathway insult follows a distinct time course and pattern (Jindahra et al., 2012; Saionz et al., 2020; Fahrenthold et al., 2021). This study showed that the LI value, which indicates the shrinkage extent of the optic tract (Bridge et al., 2011; Millington et al., 2014; Fahrenthold et al., 2021), altered distinctly compared with age-sex matched stroke-free controls. Particularly, it increased significantly in the group with a disease duration of more than 6 months. In such patients, LI was significantly associated with pRNFL thickness. Besides, both the LI and pRNFL were significantly related to the subject’s visual acuity (VA, LogMAR). To the best of our knowledge, this study is the first to explore the optic tract changes in patients with thalamic stroke and investigate their associations with retinal structural parameters by combining MRI and OCT techniques.

Previous reports showed that transsynaptic retrograde neurodegeneration (TRD) after post-thalamic visual area (occipital lobe) stroke was high after the first few years and fairly stable over the years (Cowey et al., 2011; Jindahra et al., 2012; Meier et al., 2015; Schneider et al., 2019). In our previous report (Ye et al., 2022), thalamic infarction patients with a duration of more than 6 months also showed a thinner retinal structure compared to patients with a duration of fewer than 6 months. These findings suggest that retinal degeneration follows a clear-cut time sequence, i.e., retrograde degeneration is progressive and becomes steady over time. Moreover, a well-established MRI technique has been used to reveal the detectable phenomenon of OT shrinkage in chronic hemianopia or cortical-blindness patients (Bridge et al., 2011; Millington et al., 2014). In this study, compared with controls, profound changes in LI were detected in patients with thalamic stroke, especially in patients with a duration of more than 6 months. In line with Cowey et al.’s findings (Cowey et al., 2011), the mechanism can be explained as disruption of networks within the visual tract and triggering of retrograde degeneration in the visual processing projections resulting from damage to the thalamus (particularly the lateral geniculate nucleus, LGN), a key structure involved in visual processing. Specifically, it has been suggested to lead to direct retrograde degeneration in the optic tract volume (Bridge et al., 2011; Millington et al., 2014).

Furthermore, following the TRD time course, significant correlations were found between LI and pRNFL thickness in patients with thalamic stroke with a duration of more than 6 months. Lesions involved in the visual pathway and the following retrograde degeneration may ultimately result in structural changes in the retina (Jindahra et al., 2009). Besides, large to subtle changes in the visual-related structures have been reported in patients with thalamic disorders (Agarwal et al., 2011; Pula and Yuen, 2017), and significant relationships have been found between altered retinal thickness and volumetric changes in the thalamus area (Mutlu et al., 2018). Recently, by combining structural and functional MR techniques, Conrad et al. found radiological evidence of white matter volume loss following thalamic infarction and revealed its implications for cortical areas involved in sensory and ocular function (Conrad et al., 2022). Given the fact that pRNFL (located around the optic nerve head) can characterize the global ganglion cell axonal integrity and directly connect to the brain (Saidha et al., 2013) and can represent the most proximal part of the visual projection pathway (Jimenez et al., 2014), pRNFL might be more sensitive to damage in the visual pathway. Thus, it is plausible to suggest that shrinkage of the optic tract after thalamic stroke was correlated to retinal neurodegeneration, and LI can be considered an imaging marker of neurodegeneration in thalamic stroke. Future studies with larger sample sizes of homogenous patients are needed to validate our hypothesis.

Visual complaints have been reported in patients with thalamic stroke (Roth et al., 2016; Hwang et al., 2017), implicating structural changes in the retina and the presence of axonal damage along the visual pathway. Previous reports have shown patients with thalamic stroke have vision-related problems, which impose a heavy disease burden affecting daily activities (Bogousslavsky et al., 1988; Rorden and Brett, 2000; Schmahmann, 2003; Li et al., 2018; Moon et al., 2021). Notably, we showed that LI and pRNFL thickness significantly correlated with visual acuity in patients with thalamic stroke and patients with a duration of more than 6 months. Since LI (Mehra and Moshirfar, 2022) and pRNFL (Lim et al., 2019; Rajabi et al., 2019) play significant roles in vision, it is conceivable to suggest that LI atrophy and pRNFL thinning may be associated with reduced visual acuity in patients with thalamic stroke, and they should be taken into account in treatment decisions for such patients. Apart from visual restoration training and compensatory therapy strategies, effective therapeutic options are still lacking in such patient groups (Sahraie et al., 2016; Cavanaugh and Huxlin, 2017; Fahrenthold et al., 2021). It may result from the highly individualized heterogeneity and variability that exist in the efficacy of such treatments (Saionz et al., 2020; Fahrenthold et al., 2021). Thus, it is important to accurately identify subjects who may benefit from these therapeutic options. Particularly, in addition to routine secondary prevention treatments of cerebrovascular diseases, there is still a lack of individualized treatment and evaluation indicators for stroke patients with specific and distinct clinical syndromes. It has been clarified that the existence and extent of retrograde degeneration of the neural pathway disease involved can impact interventional and natural recovery effects (Millington et al., 2014; Fahrenthold et al., 2021). This study illustrates some significant alterations in retinal structure and optic tract after thalamic stroke and explores their correlations with damage to the brain and time effects. These findings may highlight the potential of combining these two markers for disease assessment and response to therapy in these patients and suggest the need for research on related interventions.

Surprisingly, in this study, the pRNFL in patients with thalamic stroke was more sensitive to vision loss and optic tract shrinkage than the macular RNFL and GCIPL. The pRNFL is located around the optic nerve head (second cranial nerve), which plays a significant role in vision; thus, pRNFL changes may be sensitive to visual changes and *vice versa* as previously reported (Lin et al., 2018; Wagner et al., 2020). The optic tract consists of white matter microstructure (Rokem et al., 2017; Caffarra et al., 2021), which has been reported to be associated with the pRNFL thickness (Ong et al., 2015; Mutlu et al., 2017). Besides, axons from the eye to the brain leave through the optic nerve head (pRNFL) to the brain *via* the optic tract (Raz and Levin, 2014). Taken together, we suggest that pRNFL thickness in patients with thalamic stroke may be sensitive to neurodegeneration (axonal damage) and visual loss.

Our study has some limitations. First, the observational cross-sectional study design is a limitation in our study; thus, an interpretation of the causal relationship seen in our study could not be made. Second, no symptoms of the visual field or oculomotor deficits occurred, which diminished the clinical interest and expandability to some extent; however, as a type of ischemic stroke with a lower proportion, a 10-year cohort study only found out 11.7% of thalamic insulted patients who developed prominent neuro-ophthalmologic manifestations (Moon et al., 2021). Further investigations with larger samples are needed in the future. Third, we were not able to conduct a detailed analysis of the subtypes of patients with thalamic stroke (i.e., at different time points). Long-term follow-up cohort studies with data on dynamic changes are needed in the future.

## Conclusion

Shrinkage of the optic tract can be detected in patients with thalamic stroke, especially after 6 months of stroke onset. In these patients, the extent of optic tract atrophy correlated with pRNFL thickness, and they were both associated with visual acuity changes. The findings of our study emphasize the importance of further research into combining retinal imaging and MR imaging markers for disease assessment and ultimately help to guide individualized treatment choices. Longitudinal studies with a greater sample size are needed to validate our hypotheses in the future.

## Data availability statement

The raw data supporting the conclusions of this article will be made available by the authors, without undue reservation.

## Ethics statement

The studies involving human participants were reviewed and approved by the Ethics Committee of West China Hospital, Sichuan University [No. 2020(922)]. The patients/participants provided their written informed consent to participate in this study.

## Author contributions

BW: study concept and design. CY, WK, WT, JL, RP, KL, and AW: data acquisition. CY and WK: data analysis and interpretation and drafting the manuscript. ML: resources. BW: study supervision. BW and WK: critical review of the manuscript. All authors contributed to the article and approved the submitted version.

## Footnotes

1
http://www.fmrib.ox.ac.uk/fsl
