# Supplementary material for: Alterations of optic tract and retinal structure in patients after thalamic stroke
Source: Front Aging Neurosci. 2022 Jul 28;14:942438. doi: 10.3389/fnagi.2022.942438 (PMC9363922; doi:10.3389/fnagi.2022.942438)
Supplement: Supplementary file 1 [file Data_Sheet_1.docx]

**Table S1.** Ophthalmic parameters (retina microstructure and VA) between the Group 1 (≤6 months) and Group 2 (＞6 months), ipsilateral and contralateral side eye respectively.

|  | Ipsilateral eye(n=33) | | |  | Contralateral eye(n=34) | | |
| --- | --- | --- | --- | --- | --- | --- | --- |
|  | Group 1  (n=8) | Group 2  (n=25) | *P* |  | Group 1  (n=9) | Group 2  (n=25) | *P* |
| pRNFL, µm | 95.50±19.56 | 97.80±14.67 | 0.724 |  | 95.11±18.48 | 97.88±15.35 | 0.661 |
| RNFL, µm | 17.40±1.54 | 17.65±1.98 | 0.719 |  | 17.78±1.46 | 17.59±1.85 | 0.792 |
| GCIPL, µm | 61.44±8.17 | 64.52±8.58 | 0.379 |  | 62.14±7.19 | 63.80±10.64 | 0.670 |
| VA, LogMAR | 0.18±0.13 | 0.22±0.20 | 0.549 |  | 0.21±0.16 | 0.24±0.21 | 0.707 |

pRNFL: peripapillary retinal nerve fiber layer; RNFL: retinal nerve fiber layer; GCIPL: the ganglion cell and inner plexiform layer; VA: visual acuity. Group 1: ≤6 months; Group 2: ＞6 months.

**Table S2.** Comparison of optic tract parameters between thalamic stroke patients and control participants.

|  | Thalamic stroke | | | Control participants | *P* |
| --- | --- | --- | --- | --- | --- |
|  | All | Group 1(≤6 months) | Group 2(＞6 months) |  |  |
| iOT^a^, n | 189.51 ± 47.51 | 179.92 ± 44.28 | 171.77 ± 36.59 | 169.26±31.49 | 0.056^*^ |
| cOT^b^, n | 174.80 ± 39.18 | 180.46 ± 54.07 | 194.86 ± 43.63 | 167.17±31.30 | 0.437^*^ |
| LI | 0.021 (0.007 to 0.078) | 0.015 (-0.091 to 0.61) | 0.031 (0.01 to 0.135) | 0.002 (-0.006 to 0.015) | **0.011**^*^; **0.004^§^** |

^a^iOT: ipsilateral optic tract volume in patient group, right side in control group, voxel numbers; ^b^cOT: contralateral optic tract volume in patient group, left side in control group, voxel numbers; LI: lateral index. Values in bold indicate *P* < 0.05. *All thalamic stroke patients vs control participants; §Group 2 (＞6 months) vs control participants. Values in bold indicate *P* < 0.05.
